# Molecular Epidemiology of Tuberculosis in Kaohsiung City Located at Southern Taiwan, 2000-2008

**DOI:** 10.1371/journal.pone.0117061

**Published:** 2015-01-28

**Authors:** Yih-Yuan Chen, Jia-Ru Chang, Shu-Chen Kuo, Fan-Chen Tseng, Wei-Chen Huang, Tsi-Shu Huang, Yao-Shen Chen, Tzong-Shi Chiueh, Jun-Ren Sun, Ih-Jen Su, Horng-Yunn Dou

**Affiliations:** 1 National Institute of Infectious Diseases and Vaccinology, National Health Research Institutes, Zhunan, Miaoli, Taiwan; 2 Graduate Institute of Basic Medical Science, China Medical University, Taichung, Taiwan; 3 Department of Microbiology, Kaohsiung Veterans General Hospital, Kaohsiung, Taiwan; 4 Division of Clinical Pathology, Department of Pathology, Tri-Service General Hospital and National Defense Medical Center, Taipei, Taiwan; 5 Department of Internal Medicine, Ditmanson Medical Foundation Chiayi Christian Hospital, Chiaya City, Taiwan; Infectious Disease Research Institute, UNITED STATES

## Abstract

**Background:**

We present the first comprehensive analysis of *Mycobacterium tuberculosis* (MTB) isolates circulating in southern Taiwan. In this 9-year population-based study, the TB situation in the Kaohsiung region was characterized by genotypic analysis of 421 MTB isolates.

**Methods:**

All 421 isolates of MTB were analyzed by spoligotyping and MIRU-VNTR typing. Drug-resistance patterns were also analyzed.

**Results:**

The percentage of EAI (East African-Indian) strains increased across sampling years (2000–2008) in southern Taiwan, whereas the proportion of Beijing lineages remained unchanged. Clustering was more frequent with EAI genotype infections (odds ratio = 3.6, p<0.0001) when compared to Beijing genotypes. Notably, MTB resistance to streptomycin (STR) had significantly increased over time, but resistance to other antibiotics, including multidrug resistance, had not. Three major genes (*gidB*, *rpsL* and *rrs*) implicated in STR resistance were sequenced and specific mutations identified.

**Conclusions:**

This study revealed that EAI strains were highly transmissible and that STR resistance has increased between 2000 and 2008 in Kaohsiung, Taiwan.

## Background

In Taiwan, tuberculosis (TB) remains a major infectious disease [1]. It is still the leading cause of death among all communicable infectious diseases despite a steady decline in both incidence and mortality rates since 1950. Previous studies indicate that the most prevalent *Mycobacterium tuberculosis* (MTB) strains in Taiwan belong to the Beijing lineage, followed by East African-Indian (EAI) and Haarlem strains [2,3]. Notably, EAI strains in northern Taiwan comprise about 11% of MTB isolates, whereas in southern Taiwan they comprise up to 32% [4,5]. The reasons for greater EAI prevalence in southern Taiwan remain unclear, but may be related to different selection pressures.

The development of antibiotics has led to a marked reduction in TB mortality. However, antibiotic use on MTB can also select for genetic variants, and may be a factor in the epidemicity of certain MTB strains in particular regions [6]. Drug resistance in MTB is controlled by a complex genetic system involving several genes. Isoniazid (INH), streptomycin (STR), rifampicin (RIF) and ethambutol (EMB) have all been used as first-line drugs to treat TB. Resistance to INH is mediated by several genes, including *katG, inhA, kasA, ahpC* and *ndh*, and mutations in the coding or promoter regions of these genes result in complete or partial loss of gene function causing INH resistance [7,8]. Resistance to STR is mediated mainly by the *rrs, rpsL* and *gidB* genes [7,9,10]. Mutations in *rrs* and *rpsL* (encoding 16S rRNA and ribosomal protein S12, respectively) have been shown to be the basis of resistance in 60–70% of STR-resistant MTB strains [9]. The *gidB* gene (encoding 7-methylguanosine methyltransferase) is also associated with STR resistance [11,12].

We have undertaken long-term surveillance of MTB strains in southern Taiwan, including collecting information on MTB genotypes and drug-resistance phenotypes, in order to clarify associations between transmission dynamics and drug resistance. The purpose of the present study was threefold. First, we sought to determine the changes in MTB genotype frequencies over time in southern Taiwan, especially the EAI lineage, using spoligotyping and 24-loci mycobacterial interspersed repetitive unit-variable number tandem-repeat (MIRU-VNTR) technology [13]. Second, we sought to estimate the prevalence of drug-resistant MTB strains in this same sample. The proportion method for drug susceptibility testing of MTB was used, and the drugs tested were INH, STR, RIF and EMB. The cluster rates of each drug and MTB strains were also calculated to investigate the transmission dynamics of the prevalent genotypes and drug-resistant MTB strains. Third, after documenting in the present study that the prevalence of STR-resistant MTB strains in southern Taiwan increased dramatically in the past decade, we sequenced the *rrs, rpsL* and *gidB* genes to identify variants that could account for the emergent STR resistance. In conclusion, this long-term survey provides information concerning dynamic changes in genotypes, drug-resistance patterns, and cluster rates of MTB isolates in southern Taiwan.

## Methods

### Study setting

This retrospective study was conducted at the National Health Research Institutes in Taiwan. In total, 421 MTB isolates were sampled from 421 patients admitted to Kaohsiung Veterans General Hospital (KVGH) during 2000 to 2008 (S1 Table), a large medical center which handles a substantial number of TB patients referred from hospitals throughout Kaohsiung. We randomly selected 100 MTB isolates each year by choosing one isolate from each storage box. From these 100, we selected 60 isolates and subcultured them. Repeat cultures from the same patient were discarded. Archived isolates that could be re-cultured to yield sufficient material for typing and drug susceptibility testing were analyzed; in general, the number of isolates that we analyzed for each calendar year represents 4–5% of the total isolates archived per year (S2 Table). MTB isolates were confirmed by conventional methods, including routine microscopy, culture, and positive nitrate and niacin tests. All isolates were genotyped by spacer oligonucleotide typing (spoligotyping) and 24-locus MIRU-VNTR typing. The MTB strain H37Rv was used as the control. The study was approved by the Human Ethics Committee of the National Health Research Institutes, Taiwan (Code: EC1010804-E). Because of the retrospective nature, routine collection of clinical data in daily practice, and dislinkage of personal information, the requirement to obtain informed consent was waived by our institutional review board.

### DNA extraction and sequencing

Mycobacterial chromosomal DNA was extracted by boiling a cultured cell suspension scraped from Lowenstein-Jensen slants in 200 μl distilled water at 85°C for 30 min. After centrifugation, the supernatants containing DNA were removed and stored at −20°C until further use. The sequences of the primers used in this study are listed below. A 306-bp DNA fragment of the *M. tuberculosis rpsL* gene was amplified using primers *rpsL*F 5′-CCAACCATCCAGCAGCT-3′ and *rpsL*R 5′-ATCCAGCGAACCGCGGA-3′. 238-bp and 240-bp DNA fragments of the *M. tuberculosis rrs* gene were amplified using two primers sets: *rrs*238F 5′-GATGACGGCCTTCGGGTT-3′ and *rrs*238R 5′-TCTAGTCTGCCCGTATCG-3′; *rrs*240F 5′-GTAGTCCACGCCGTAAA-3′, *rrs*240R 5′-AGGCCACAAGGGAACG-3′. A 675-bp DNA fragment of the *M. tuberculosis gidB* gene was amplified using the primer pair *gidB*F 5′ GTCCCTCCACTCGCCATC3′ and *gidB*R 5′GCGGAGTGCGTAATGTCTC3′.

### Spoligotyping and spoligotype analysis

Spoligotyping was carried out according to the manufacturer’s instructions (Isogen Bioscience B.V., Maarsen, The Netherlands). The resulting spoligotypes were documented using a binary code representing either a positive or a negative hybridization result (n and o, respectively) and analyzed using Excel software for grouping and ordering the patterns. The SpolDB4 database [14] and a web-based computer algorithm, Spotclust [15], were used to assign new isolates to families, subfamilies and variants. SpolDB4-assigned names (shared types) were used whenever a spoligopattern was found in the database. Patterns not found in SpolDB4 were assigned to families and subfamilies by Spotclust. Spoligotypes described only once (non-clustered) in this study and in spolDB4 were designated as “orphan”. A cluster was defined as two or more isolates from different patients with identical spoligotype and MIRU-VNTR patterns.

### 24-locus MIRU-VNTR typing

The 12 classical MIRU-VNTR loci (‘12-locus’), 3 exact tandem repeats (ETR A, B and C) and 9 additional loci (Mtub04, Mtub21, Mtub29, Mtub30, Mtub34, Mtub39, QUB11b, QUB26 and QUB4156) were selected and individually amplified in all MTB isolates as previously described by Supply et al. [16]. The resulting typing pattern from the 24 loci was used to create a 24-digit allelic profile for each isolate.

### Drug susceptibility test

Drug susceptibility testing was performed according to the Clinical and Laboratory Standards Institute (CLSI) standard [17]. The tests were conducted by using the agar proportion method utilizing Middlebrook 7H10 agar supplemented individually with the following drugs: EMB (5 and 10 μg/ml), INH (0.2 and 1 μg/ml), RIF (1 μg/ml) and STR (2 and 10 μg/ml).

### Statistical analysis

All MTB isolates based on the 24-loci typing result, numbers of total (T), unique (U) and clustered isolates (C) and clusters (N) were tabulated. Transmissibility rate (R% = (C–N)/T) was calculated as previously defined [17]. Distributions of MTB genetic lineages and clusters in relationship to percentages of drug resistance, time trends (in every 1- or 3-year interval) and other characteristics were examined by Fisher’s exact test and by the Cochran-Armitage trend test. Odds ratio (OR) with 95% confidence interval (95% C. I.) was used to express the magnitude of associations using the most frequent group as the reference in the analysis. In analyses of time trends, statistical tests were repeated by substituting every year with every 3-years to avoid biases due to small sample numbers in some years, and only results with consistent significance and trends in both time intervals were reported.

## Results

### Dynamics of *M. tuberculosis* genotypes in southern Taiwan, 2000–2008

**Figure 1:**
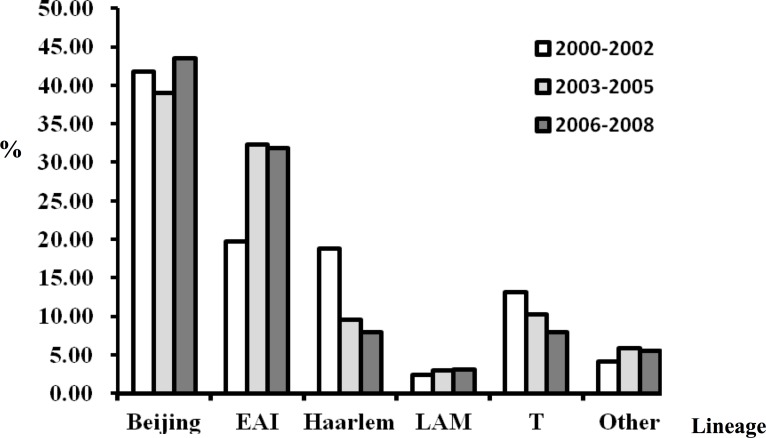
Overview of the dynamic changes in genotype frequencies in all MTB strains isolated from 421 patients.

In total, 421 MTB isolates collected between 2000 and 2008 at Kaohsiung Veterans General Hospital from 421 patients with culture-confirmed TB were subjected to spoligotyping and MIRU-VNTR typing. Of the 421 clinical isolates analyzed, the most prevalent genotypes were Beijing, identified in 176 isolates (41.8%), followed by EAI (120/421; 28.5%), Haarlem (49/421; 11.6%), T (43/421; 10.2%), and LAM (12/421; 2.9%) (Table 1). The Beijing lineage, the most prevalent genotype in Taiwan, represented 28.3–50% of the MTB isolates annually among the samples we examined. Annual proportions of the EAI genotype fluctuated between 8.3–41.8%, the Haarlem genotype between 4.8–22.7%, and the T genotype between 1.8–20.8%. There was a decreasing trend for the Haarlem genotype (p trend = 0.01); no significant trends were found for the other genotypes (Fig. 1).

**Table 1:** Genotype distribution based on combined spoligotyping and MIRU-VNTR typing of MTB isolates from culture-positive TB patients in southern Taiwan, 2000–2008.

	**No. of isolates (%)**									
	**2000**	**2001**	**2002**	**2003**	**2004**	**2005**	**2006**	**2007**	**2008**	**Total**
**Beijing**	12	19	20	13	20	21	24	25	22	176 (41.8)
**EAI**	2	9	13	19	13	12	13	16	23	120 (28.5)
**Haarlem**	3	10	10	5	2	6	5	4	4	49 (11.6)
**LAM**	1	1	1	1	0	3	3	1	1	12 (2.9)
**T**	5	2	9	7	5	2	6	6	1	43 (10.2)
**Other**	1	3	1	1	2	4	4	1	4	21 (5)
**Total**	24	44	54	46	42	48	55	53	55	421 (100)

### Increased resistance to STR across sampling years

**Figure 2:**
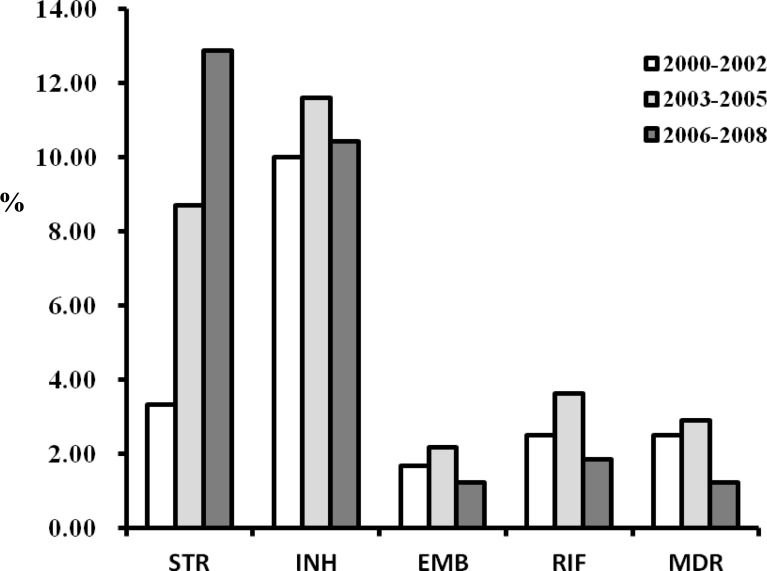
Overview of the dynamic changes in drug-resistance patterns in all MTB strains isolated from 421 patients.

Of the 421 strains isolated across sampling years, nearly 84.8% (357/421) were sensitive to all four of the first-line agents tested, 8.8% (37/421) were resistant to STR, 10.7% (45/421) were resistant to INH, 1.7% (7/421) were resistant to EMB, 2.6% (11/421) were resistant to RIF, and 2.1% (9/421) were multidrug resistant (MDR) (S1 Table). In terms of changes over time, resistance to STR increased significantly during the sampling period (p-trend = 0.003), but resistance to the other antibiotics tested, including MDR, did not change significantly (Fig. 2). We further examined independent risk factors for drug resistance by multivariable logistic regression. Dummy variables were coded for genotypes Beijing, EAI, Haarlem and T, and the remaining genotypes were grouped as “others”. Year of strain isolation was represented as a continuous variable at 1- or 3-year intervals, and strains were dichotomized into “clustered” or not based on 24-loci MIRU-VNTR typing. For STR resistance, higher risks were independently associated with later years (p = 0.007); no independent risk factor was found for INH resistance.

### Associations between strain clustering rate and *M. tuberculosis* genotype

To better understand relationships between MTB genotypes and antibiotic resistance involved in transmission fitness, we first calculated the clustering rate of each genotype (Table 2). When using the most prevalent genotype, Beijing, as the reference group, clustering was more frequent with EAI genotype infections (odds ratio (OR) = 3.6 (95% C.I. = 2.21–5.87; exact p <0.0001)). The EAI genotype showed significantly higher transmissibility (47.5%) than Beijing (20.0%) (Table 2), whereas the T (0%) lineages showed significantly lower transmissibility when compared with the total population (23.0%) (Table 2).

**Table 2:** Univariate analysis of cluster rates in different MTB lineages.

	**No. of patients**	**Patients in clusters (%)**	**Strain-clustering rate (N-1; %)**	**OR**	**95% CI**	**P value**
**Total**	421	136 (32.3)	23.0			
**Beijing**	176	53 (30.1)	19.9	reference		
**EAI**	120	73 (60.8)	47.5	3.6046	2.2129 − 5.8716	<0.0001
**Haarlem**	49	8 (16.3)	8.2	0.4528	0.1988 − 1.0314	0.07
**T**	43	0	0	NA		
**Others**	33	2 (6.1)	3.0	0.1497	0.0346 − 0.6484	0.003

### Mutation of *gidB* and *rpsL* associated with STR resistance level

To investigate possible reasons for the increased rate of STR resistance across sampling years, all STR-resistant strains were analyzed in more detail. However, we were unable to get sequence information for two strains. Of the 35 STR strains examined in this study, 15 isolates (42.9%) were resistant to the lower concentration of STR (2 μg/ml) and 20 (57.1%) were resistant to the higher concentration (10 μg/ml). Furthermore, 20 (57.1%) were resistant to at least one additional drug (INH, RIF, EMB), and 2 (5.7%) were MDR (resistant to at least INH and RIF). Based on the defined spoligotypes, the most frequent STR-resistant strains belong to the Beijing lineage (20/35; 57.1%), followed by EAI (8/35; 22.9%), T (5/35; 14.2%), Haarlem (1/35; 2.9%) and LAM (1/35; 2.9%).

To determine whether mutation of the *rpsL, rrs* and *gidB* genes could contribute to the STR-resistance phenotype, these three genes in all 35 STR isolates were sequenced and classified according to the level of STR resistance (Table 3). Of the 15 low-level STR-resistant strains, 12 (80%) carried a nonsynonymous mutation in *gidB* (Table 3). Surprisingly, no mutation was found in the *rpsL* gene in these low-level STR-resistant MTB strains (Table 3). These results suggest that low-level STR resistance might be caused by mutation of the *gidB* gene. However, the existence of 2 low-level STR-resistant strains showing no mutation in any of *gidB, rpsL* and *rrs* suggests there must also be other mechanisms for STR resistance.

**Table 3:** Mutations in the *gidB*, *rps*L and *rrs* genes in streptomycin-resistant MTB strains.

**Phenotypic resistance**	**Mutation found in:**			**No. of isolates**
	***GidB***	***rpsL***	***rrS***	
**S_L_^a^**				
	E92D; A205A^b^	W^c^	513 A-C^d^	2
	E92D; A205A; L137P	W	W	2
	W	W	W	2
**PS_L_^e^**				
	38frameshift	W	W	1
	P75S	W	W	1
	E92Stop	W	W	1
	M178R	W	W	1
	E92D; A205A	W	W	1
	E92D; A205A	W	513 A-C	1
	E92D; A205A; 38frameshift^f^	W	W	1
	V110V; A205A	W	W	1
	V110V; A205A; F12C	W	W	1
**S_H_^g^**				
	E92D; A205A	W	W	1
	E92D; A205A	K43R	W	3
	E92D; A205A; A10V	K43R	W	1
	V110V; A205A	W	513 A-C	1
	V110V; A205A	K88R	W	1
	V110V; A205A; R20R	K88R	W	1
	V110V; A205A; T146T	K43R	W	1
**PS_H_^h^**
	E92D; A205A	W	513 A-C	1
	E92D; A205A	K15N^c^	513 A-C	1
	E92D; A205A	K43R	W	4
	E92D; A205A	K88R	W	1
	W	K88T	W	1
	W	W	513 A-C	2
	W	W	W	1
**Total**				35

^a^: Low-level streptomycin resistant (2 μg/ml)

^b^: Symbols (amino acid); V110V and A205A are synonymous mutations and are not expected to affect the functionof the *gidB* protein

^c^: The sequence of the clinical isolate was 100% identical to that of H37Rv.

^d^: Symbols (nucleotide)

^e^: Low-level streptomycin resistant with additional resistance to at least one of INH, EMB and RIF.

^f^: delC115 (nucleotide)

^g^: High-level streptomycin resistant (10 μg/ml)

^h^: High-level streptomycin resistant and resistant to at least one of INH, EMB and RIF.

Of the 20 high-level STR-resistant strains, 14 (70.0%) were found to carry a nonsynonymous mutation in the *gidB* gene and 16 were found to have a nonsynonymous mutation in the *rpsL* gene (Table 3). Among the 14 *rpsL* mutants the most frequent mutation was K43R (9/14; 64.3%), followed by K88R (3/14; 21.4%) (Table 3). The most frequently paired *gidB* and *rpsL* double mutants were E92D + A205A (*gidB*) and K43R (*rpsL*) (8/20; 40.0%), followed by V110V + A205A (*gidB*) and K88R (*rpsL*) (2/20; 10.0%), V110V + A205A (*gidB*) and K43R (*rpsL*) (1/20; 5.0%) and E92D + A205A (*gidB*) and K88R (*rpsL*) (1/20; 5.0%) (Table 3). Only one high-level STR-resistant strain (1/20; 5.0%) had no mutations in any of the three genes examined.

Mutation of the *rrs* gene was observed at similar frequencies in both high- (3/15; 20.0%) and low-level (5/20; 25.0%) STR-resistant strains (Table 3).

### Lineage-specific polymorphisms in Beijing and EAI lineages

Based on spoligotyping and sequencing results, the types and frequencies of mutations in the *gidB, rpsL* and *rrs* genes in each STR-resistant MTB lineage were categorized. Of the 35 isolates, 77.1% (27/35) carried nonsynonymous mutations in the *gidB* gene and 40.0% (14/35) in the *rpsL* gene; and 22.9% (8/35) had an A→C transversion in the *rrs* gene (Table 4). Among the Beijing strains, the percentages of strains carrying nonsynonymous mutations in *gidB* and *rpsL* were 90.0% (18/20) and 45.0% (9/20), respectively; and 30% (6/20) carried an A→C transversion in *rrs* (Table 4). The percentages of EAI strains carrying nonsynonymous mutations in *gidB* and *rpsL* were 37.5% (3/8) and 50.0% (4/8), respectively; and 12.5% (1/8) carried an A→C transversion in *rrs* (Table 4). Mutations in *gidB* and *rrs* occurred at significantly higher frequencies in Beijing strains compared to both the total isolates and EAI isolates.

**Table 4:** Mutation frequencies of *gidB*, *rps*L and *rrs* genes in different MTBlineages.

**Lineages**	***gidB***	**F(%)^a^**	***rpsL***	**F(%)**	***rrs***	**F(%)**
**Beijing**	E92D; A205A^b^	14(40.0)	K15N^c^	1(2.9)	513A-C^d^	6(17.1)
	E92D; A205A; A10V	1(2.9)	K43R	7(20.0)	W	14(40.0)
	E92D; A205A; L137P	2(5.7)	K88R	1(2.9)		
	E92D; A205A; 38frameshift^e^	1(2.9)	W	11(31.4)		
	W	2(5.7)				
**EAI**	V110V; A205A	4(11.4)	K43R	2(5.7)	513A-C	1(2.9)
	V110V; A205A; F12C	1(2.9)	K88R	2(5.7)	W	7(20.0)
	V110V; A205A; R20R	1(2.9)	W	4(11.4)		
	V110V; A205A; L49R	1(2.9)				
	W	1(2.9)				
**T**	38frameshift^e^	1(2.9)	W	5(14.3)	513A-C	1(2.9)
	E92Stop	1(2.9)			W	4(11.4)
	M178R	1(2.9)				
	W	2(5.7)				
**Haarlem**	W	1(2.9)	K88T	1(2.9)	W	1(2.9)
**LAM**	P75S	1(2.9)	W	1(2.9)	W	1(2.9)
**Total**		35(100)		35(100)		35(100)

^a^: Frequency (no. of isolates/total streptomycin-resistant strains (n = 35))

^b;c^: Symbols (amino acid); V110V and A205A are synonymous mutations and are not expected to affect the function of the *gidB* protein

^d^: Symbols (nucleotide)

^e^: delC115 (nucleotide)

Surprisingly, lineage-specific polymorphisms were found in both Beijing and EAI strains. In the *gidB* gene, most mutated Beijing strains carried nonsynonymous E92D (GAA to GAC) and synonymous A205A (GCA to GCG) mutations, whereas the EAI lineage carried synonymous V110V (GTG to GTT) and synonymous A205A (GCA to GCG) mutations (Table 4). However, lineage-specific polymorphisms were not observed in the T strains. Nonsynonymous mutations affecting codons 43 (K43R; AAG to AGG) and 88 (K88R; AAG to AGG) of the *rpsL* gene were found in both the Beijing and EAI strains, but not in the other strains (Table 4).

## Discussion

The present study revealed the percentage of EAI lineages in Kaoshiung in southern Taiwan to have increased (19.7% to 31.9% of cases) across sampling years (2000–2008), whereas the percentage of Beijing strains remained stable (41.8% to 43.6% of cases). The Beijing genotype overall was the most frequent genotype identified in southern Taiwan. This result coincides with those of previous studies in Taiwan and globally, in which Beijing genotypes are usually the most prevalent MTB strains and are often associated with major TB outbreaks [2,18,19,20]. However, the Beijing strains in southern Taiwan (41.8% of cases; Table 1) constitute a smaller percentage compared to what we previously observed in northern Taiwan (52.5% of cases) (p<0.05) [5]. Notably, EAI strains in southern Taiwan comprise up to 28.5% of all MTB strains sampled there (Table 1), but in northern Taiwan they comprise only about 11% [5]. The EAI lineage is more prevalent in Southeast Asia, particularly in the Philippines (73%), in Myanmar and Malaysia (53%), and in Vietnam and Thailand (32%) [15]. It is a very interesting question why southern Taiwan has a significantly higher percentage of EAI strains than northern Taiwan. As EAI strains are highly prevalent in nearby countries, their high prevalence in southern Taiwan might be due to frequent travel and immigration of infected individuals from these countries in the last decade. The proportions of Haarlem, T, and LAM lineages were 11.6%, 10.2%, and 2.9%, respectively (Table 1). A similar distribution was also reported by Huang et al. in southern Taiwan (Haarlem strains, 13%; T strains, 6%, collected from Tainan Chest Hospital and Kaohsiung Medical University Hospital) [21].

To investigate relationships between transmission and genotype of MTB strains, we analyzed the cluster rates in each MTB lineage. Our results show that the transmission of EAI strains (47.5%; OR 3.6) is significantly higher than that of Beijing (20.0%) and other strains in southern Taiwan (Haarlem strains, 8.2%; T strains, 0%) (Table 2). Lower cluster rates could be due to the low sampling number of Haarlem and T strains in this study (Fig. 1). Conversely, the high cluster rate observed for EAI strains could contribute to their increased representation.

MTB genotype fitness is determined by complex factors, including bacteria and host interactions [22,23,24,25,26]. Adaptive pressures from antibiotics, for example, can lead to selection of resistant bacteria. In the 9-year surveillance of MTB strains reported in the present study, frequent resistance was detected to the first-line anti-TB drugs INH (10.7% of cases; 45/421) and STR (8.8% of cases; 37/421) (S1 Table). From 1996 to 2002, primary resistance to INH in Taiwan rose from 4.7% to 12%, as reported by Hsueh et al. [27]. Over the same period, primary resistance to STR increased from 4% to 11%, and the percentage of cases resistant to EMB (0.7–5.9%) or RIF (1–6%) was significantly lower than for STR (5–11%) or INH (5–12%) [27]. Our observations in the present study are similar to those of Hsueh et al. [25]: the percentage of isolates resistant to STR or INH was significantly higher than for EMB or RIF. Analysis of the cluster rates in each drug-resistant strain revealed that only INH- and STR-resistant strains displayed transmissibility (8.9% and 10.8%, respectively a cluster is defined based on similarity and is assumed to have been the result of recent transmission); no transmissibility was found in the case of EMB—or RIF-resistant strains. This may explain why the percentages of INH- and STR-resistant MTB strains were higher than those of EMB- and RIF-resistant MTB strains across sampling years.

In further characterization of the STR-resistant MTB strains, three major resistance genes (*gidB, rpsL, rss*) were sequenced. In our cases, MTB strains showing resistance to STR and having a nonsynonymous mutation at codon 43 (K43R) or codon 88 (K88R) of the *rpsL* gene were all resistant to a high concentration of STR (10 μg/ml) (Table 3). The proportion of strains carrying the K43R mutation (9/35, 25.7%) was significantly higher than the proportion carrying K88R (3/35, 8.6%). A similar trend was also reported by Nhu et al. among TB isolates in Vietnam [28]. The existence of lineage-specific polymorphisms of the *gidB* gene in Beijing (E92D and A205A) and EAI (V110V and A205A) genotypes was also described by Feuerriegel et al. and Spies et al. [7,9]. Our results suggest that *gidB* variants are largely responsible for low-level STR resistance, and *rpsL* variants for high-level STR resistance. The roles of *gidB* and *rpsL* in resistance to STR have previously been described [7,11,28].

In conclusion, this multi-year study identified dynamic changes of MTB strains in southern Taiwan. EAI strains were found to be transmissible. In addition, the types and frequencies of *gidB, rpsL and rss* gene variants in STR-resistant MTB strains were determined. The transmissibility of the EAI genotype in southern Taiwan should be considered in control policy. Taken together, this study revealed that EA1 strains were more transmissible and that STR resistance has increased between 2000 and 2008 in Kaohsiung, Taiwan.

## Supporting Information

S1 Table Drug susceptibility and resistance to first-line anti-tuberculosis drugs. (DOCX)

S2 Table MTB isoates from 2000–2008 archived at −80°C in the KGVH laboratory. (DOCX)
