# Supplementary material for: Molecular Epidemiology of Tuberculosis in Kaohsiung City Located at Southern Taiwan, 2000-2008
Source: PLoS One. 2015 Jan 28;10(1):e0117061. doi: 10.1371/journal.pone.0117061 (PMC4309396; doi:10.1371/journal.pone.0117061)
Supplement: S1 Table — (DOCX) [file pone.0117061.s001.docx]

**Supplementary Table S1. Drug susceptibility and resistance to first-line anti-tuberculosis drugs**

|  | **No. of MTB isolates** | | | | | | | | | |
| --- | --- | --- | --- | --- | --- | --- | --- | --- | --- | --- |
|  | **2000** | **2001** | **2002** | **2003** | **2004** | **2005** | **2006** | **2007** | **2008** | **Total** |
| **STR** | 1 | 1 | 2 | 2 | 6 | 4 | 7 | 4 | 10 | 37 |
| **INH** | 0 | 5 | 7 | 3 | 9 | 4 | 5 | 1 | 11 | 45 |
| **EMB** | 0 | 2 | 0 | 0 | 1 | 2 | 0 | 0 | 2 | 7 |
| **RIF** | 0 | 1 | 2 | 1 | 4 | 0 | 1 | 0 | 2 | 11 |
| **Any R** | 1 | 6 | 7 | 4 | 12 | 5 | 9 | 4 | 16 | 64 |
| **All S** | 23 | 38 | 47 | 42 | 30 | 43 | 46 | 49 | 39 | 357 |
| **MDR** | 0 | 1 | 2 | 1 | 3 | 0 | 0 | 0 | 2 | 9 |

STR: Streptomycin; INH: Isoniazid; EMB: Ethambutol; RIF: Rifampicin; Any R: Resistant to one of the drugs; All S: Susceptible to all of the drugs tested; MDR: resistant to at least INH and RIF
