# Supplementary material for: Molecular Epidemiology of Tuberculosis in Kaohsiung City Located at Southern Taiwan, 2000-2008
Source: PLoS One. 2015 Jan 28;10(1):e0117061. doi: 10.1371/journal.pone.0117061 (PMC4309396; doi:10.1371/journal.pone.0117061)
Supplement: S2 Table — (DOCX) [file pone.0117061.s002.docx]

**Supplementary Table S2. MTB isoates from 2000-2008 archived at -80^o^C in the KGVH laboratory**

| Year | Total archived isolates (no.) | Isolates (no.) included in this study |
| --- | --- | --- |
| 2000 | 1084 | 24^a^ |
| 2001 | 1005 | 44 |
| 2002 | 1136 | 54 |
| 2003 | 1043 | 46 |
| 2004 | 1038 | 42 |
| 2005 | 1272 | 48 |
| 2006 | 995 | 55 |
| 2007 | 1210 | 53 |
| 2008 | 1022 | 55 |
| Total | 9805 | 421 |

^a^The lower rate of successful re-culture in calendar year 2000 may be related to renovation of the TB laboratory that took place in that year.
